# Strengthening Digital Transformation and Innovation in the Health Care System: Protocol for the Design and Implementation of a Multidisciplinary National Health Innovation Research School

**DOI:** 10.2196/46595

**Published:** 2023-05-31

**Authors:** Jens M Nygren, Lina Lundgren, Ingela Bäckström, Petra Svedberg

**Affiliations:** 1 School of Health and Welfare Halmstad University Halmstad Sweden; 2 School of Business, Innovation and Sustainability Halmstad University Halmstad Sweden; 3 Department of Communication, Quality Management and Information Systems Mid Sweden University Östersund Sweden

**Keywords:** digital health technology, doctoral education, health, health care, health innovation, implementation, improvement, innovation, research school

## Abstract

**Background:**

Digital health technologies have the potential to transform health care services to be more cost-effective, coordinated, and accessible on equal terms for entire populations. In the future, people will be assisted by such technologies to monitor their health status, take preventive measures, and have more control of their health situation. An increase in digital supplementation or substitution of physical care visits can potentially add value to patients and care providers by increasing accessibility, safety, and quality of care. However, health care organizations struggle with the challenges of developing and implementing digital health technologies and services in practice. As a response to this, we have developed a national multidisciplinary research school to increase competence and capacity for research on the development, implementation, and dissemination of digital health technology solutions. The overall aim of the research school is to increase national competence and capacity for the development, implementation, and dissemination of digital health technology to increase the preparedness to support and facilitate the ongoing digital transformation in the health care system.

**Objective:**

The purpose of this paper is to outline the protocol for the development and implementation of a national multidisciplinary doctoral education program of health innovation supporting digital transformation in the health care system.

**Methods:**

A national multidisciplinary research school for health innovation was planned in collaboration between 7 Swedish universities and their partners from industry and the public sector. The research school will run over 6 years, of which 5 years are dedicated for the doctoral education program and 1 year for the project start-up and closing. In this paper, we outline the methodological approach of the research school; the combining of knowledge and expertise of the universities that are important to run the research school; the jointly formulated research-oriented and societally relevant research focus, goals, and objectives for the research school; the established and developed relationships with partners from industry and the public sector for joint research training projects; the forms of collaboration in the research school; and the format of the doctoral education process.

**Results:**

The research school was funded in December 2021 and started in March 2022. The research school starts with an initiation period from March 2022 to December 2022 where the infrastructure and the action plans to run the school are set up. The PhD projects start in January 2023, and these projects will be completed in 5 years. Additional activities within the research program are doctoral courses, networking activities, and dissemination of results.

**Conclusions:**

The network of several partners from industry, public sector, and academia enables the research school to pose research questions that can contribute to solving relevant societal problems related to the development, evaluation, implementation, and dissemination of methods and processes assisted by digital technologies. Ultimately, this will promote innovation to improve health outcomes, quality of care, and prioritizations of resources.

**International Registered Report Identifier (IRRID):**

PRR1-10.2196/46595

## Introduction

### Background

Health care systems around the globe are facing major challenges and need to adapt to changes in demographics and patient needs, transitions from hospital care to home care, limitations in recourses, and requirements for reducing costs while maintaining and improving the quality of care [1,2]. One of the most important resolutions to support and facilitate this ongoing transformation of the health care system is through digitalization [3,4]. Advances in digital health technologies could both reduce costs and revolutionize health care. However, the uptake of health and welfare technology is often slow and with variable results [5]. Thus, the success of digitalization places great demands on the health care system and requires specific competences and close collaboration with industry and universities.

The Sustainable Development Goals for health and well-being lay out ambitious targets for disease reduction and health equity for 2030 [6]. Health systems are highly labor intensive, and health care providers and professionals play a key role in transforming the health care system to achieve these goals. However, an effective health care delivery system also depends on the coproduction with patients [7,8]. In Sweden, the health care reform *God och nära vård* (Good and close care) emphasizes the role of the patients as an active agent in their own care and the health care systems need to revise the focus of care processes, from organizational perspectives to the care pathways of patients [9]. Swedish health care has historically invested in emergency hospitals and specialist care rather than primary care and home care. This has resulted in a health care system that, in international comparison, is of high quality in terms of medical results, but has poor results in continuity, patient participation, and accessibility [9-11]. Thus, a transition of focus of care from organization to patient-provider relationship perspectives, from being reactive to proactive, from being fragmented to coherent, and from seeing patients as passive recipients to becoming active actors is desirable [9]. To be able to respond to these new demands and expectations, educational, research, and innovation initiatives need to address the ongoing digital transformation of health care, to ensure health care professionals can make use of such opportunities [12,13].

The definition and meaning of digitalization are broad and can, in the health care context, be understood as the use of a variety of converging digital tools and systems, such as wireless sensors, information systems, social networking, and mobile connectivity in health-related products and services, that is, a technology-based intervention that aims at maintaining or promoting health, well-being, quality of life, or increasing efficiency in the service delivery system of welfare, social, and health care services, while improving working conditions of the staff [14]. Digital health technologies will, in the future, increasingly enable people to better monitor their health status, take preventive measures, and take more control of their health situation. Such supplementation or substitution to physical care visits can potentially create value to patients and care providers by increasing accessibility, safety, and quality of care [15]. However, many health care systems struggle with the aspiration to stimulate the development and implementation of digital health technologies and services, as part of an undertaking to improve and evaluate health system performance [16,17]. A major challenge when it comes to the use of digital innovations for the transformation of health care is the dissemination and implementation in practice and to make sure it results in actual improvement and value for patients and the health care system [18,19]. The prolonged time for translating research into practice needs attention and improvements to be based on increased influence from patients, clients, and practitioners [20]. There is scarce evidence about how digital health technologies are implemented in health care practice and how they improve health care and health outcomes in patients [18,21]. Given this context, there is a need for additional educational offerings and research about the benefits of the process of digitalization of health services and how they outweigh efforts made and investments in costs [22]. This points to a need to advance theory and empirical evidence on coproduction requirements of digital technologies in the health care sector and opportunities to bring together insights from research on technological development and existing knowledge about innovation, implementation, and improvement science.

As a response to this challenge, we have developed and recently established a national multidisciplinary research school for health innovation in collaboration between 7 Swedish universities and their partners from the industrial and public sectors. The ambition of the research school is to strengthen the national capacity for health care improvement through digital health innovation and to significantly increase competence in the industrial and public sectors. Through enhanced collaboration across academic, industrial, and public sector partners, the overall purpose of this research school is to increase national competence and capacity for the development, implementation, and dissemination of digital health technology to increase the preparedness to support and facilitate the ongoing digital transformation in the health care system.

The intention of the research school is for all partners to benefit from each of the specializations, positions, and expertise, to create a multidisciplinary learning environment with added value for the doctoral students, the involved researchers, and partners. Through this environment, the participating partners will develop expert competence in areas directly relevant for their business and increased capacity for research and development in the long term. Additionally, access to and broadening of already existing networks and international collaborations will increase.

### Aim

The purpose of this paper is to outline the protocol for the development and implementation of a national multidisciplinary doctoral education program of health innovation supporting digital transformation in the health care system.

## Methods

### Research School Approach

The research school will use a coproduction approach [23] as a theoretical and methodological starting point. Coproduction is proposed as a means to deal with different forms of uncertainty and the complex nature of problems, both in the present and in the future. The approach is value driven and built on principles of shared power and reciprocal exchange between diverse stakeholders. Coproduction processes can harness the synergistic effect of collaborations and offer insights into experience, inspiration, values, goals, and shared vision among stakeholders. This project further involves creating permanent structures for coproduction through building a network for communication of results and impacts, strengthening translation of the knowledge generated in the research school into practice.

A collaboration network of universities, companies, and public sector organizations in Sweden was established during 2019-2021 (prephase) to create a closer connection between research, higher education, health services, and industry through the formation of a research school. The process started and formed the basis for a 2-year coproduction prephase that resulted in a structure and guidelines for running the research school. During the 2-year coproduction prephase, the 7 universities met biweekly, with workshops and meetings being held with companies, regions, and municipalities in several stages to explore what values were of importance for them. This prephase also aimed to narrow down which collaborators were potential participants in the research school. Letters of intent were written with those who were interested in joining the research school, and at a later stage final agreements were written.

The research school runs over 6 years (between 2022 and 2027), of which 5 years are dedicated for the doctoral education program and 1 year for the project start-up and closing. In the following section, we outline the approach and structure of the research school. First, we present the combining of knowledge and expertise of the universities that are important to run the research school. Second, we describe the jointly formulated, research-oriented, and societally relevant research focus, goals, and objectives for the research school. Third, we describe the established and developed relationships with partners from industry and the public sector for joint research training projects. Finally, we present the forms of collaboration in the research school and the format of the doctoral education process.

### Combining of Knowledge and Expertise From the Universities to Run the Research School

The 7 universities committed to the research school formation all have research specializations related to the area of digitalization in health care, in different academic disciplines. The 7 universities together have 16 doctoral education programs in different subject areas related to the intention of the research school (Table 1); 9 of these doctoral programs are connected to the theme “Supporting real-world implementation,” 4 are connected to the theme “Developing valuable digital technologies,” and 3 are connected to the theme “Coordinating and optimizing the innovation process.” Some of the universities have doctoral education programs in more than 1 subject area. Accordingly, the universities contribute to the research school with doctoral education programs in different subject areas which, based on both similarities and differences, create unique perspectives to the 3 overall themes in the research school. This will make the scope of the research school broad and allow for cutting-edge research on different multidisciplinary aspects of health innovation. An important value is that the universities contribute to lectures, seminars, and supervision held by leading national researchers. Thus, the 7 universities collaborating on this effort are, in terms of areas of strength, both supporting and complementing each other.

**Table 1:** Contributions and scientific position of the participating universities.

Participating university/institute	Subject areas	Theme contributions^a^	Research specializations	Research initiatives and centers	Innovations centers^b^
Blekinge Institute of Technology	Applied health technology	Technologies	Gerontechnology	SNAC^c^	Health Technology Research Lab
Halmstad University	Information technology, innovation science, health, and lifestyle	Technologies, implementation, and innovation	Health innovation and information-driven care	RFI^d^, CAISR Health^e^, and IDC^f^	Leap for life
Jönköping University	Health and care science, welfare and social science, disability research	Implementation	Quality improvement, innovation, and leadership in health and welfare	Jönköping Academy for Improvement in Health and Welfare	—^g^
Mid Sweden university	Health science, quality management	Implementation	Rehabilitation science and quality technology and management	—	—
Mälardalen University	Health and welfare, embedded systems, innovation, and product realization	Implementation and innovation	Medical and health technology for diagnosis and prevention	Embedded sensor systems for health plus	—
University of Skövde	Health science, information technology	Technologies and implementation	Health in the digital society	INFINIT^h^ and DHEAR^i^	—
University West	Work-integrated and workplace learning, production technology	Implementation and innovation	Work-integrated learning	LINA^j^ and LOV^k^	Health Academy West

^a^Technologies (Developing valuable digital health technologies), implementation (Supporting real-world implementation), innovation (Coordinating and optimizing the innovation process).

^b^The 7 universities have a history of research and education in complementary areas relating to health innovation. A distinguishing feature of this research and education is a high degree of collaboration and coproduction with industries and public sectors and strategic ambitions that combine goal setting for achieving both scientific excellence and value creation in society. Each university has specific research areas and specializations that position them nationally and internationally, and that contribute in different ways with relevant and specialized input to the 3 research themes of the research school (Table 2). The intention of the research school is to benefit from each of the specializations, positions, and expertise, and thereby create a multidisciplinary learning environment with added value for the doctoral students, the involved researchers, and partners.

^c^SNAC: Swedish National Study on Ageing and Care.

^d^RFI: research for innovation.

^e^CAISR Health: Center for Applied Intelligent Systems Research Health.

^f^IDC: information-driven care.

^g^Data not available.

^h^INFINIT: Research directed towards the generation, quality assurance, analysis and visualization of data using information technology systems and models.

^i^DHEAR: Digital Health Research.

^j^LINA: learning in and for the new working life.

^k^LOV: learning and caring for sustainable health.

**Table 2:** Themes of health innovation for successful transformation of the health care system.

Theme	Focus
Developing valuable digital technologies	Focus on the coproduction of digital technologies that address the needs of stakeholders and users, and can be adopted easily in practice
Supporting real world implementation	Focus on the coproduction of theoretical and applied knowledge on challenges and opportunities of implementing digital technologies in health and welfare
Optimizing adoption and diffusion of health innovations	Focus on the coproduction of improvement processes in health and welfare transformation

### Research Focus, Goals, and Objectives for the Research School

In designing the research school, a multidisciplinary approach is taken to integrate theoretical, empirical, and experiential knowledge from the 16 doctoral education programs at the 7 participating universities relating to the entire health innovation pathway. By combining the 16 doctoral education programs the aim is to integrate research of different content, from different perspectives, supported by unique specializations and methodological competencies. Based on several discussions and multidisciplinary workshops between the universities, 3 overarching and interdependent themes of digital innovation for successful transformation of the health care system are defined (Table 2) for the research school: (1) Developing valuable digital health technologies, (2) Supporting real-world implementation, and (3) Coordinating and optimizing the innovation process (Table 2).

The studies within the themes will focus on 6 identified overarching research questions to which the doctoral projects relate and contribute with new knowledge:

- How health innovations can be developed, implemented, and evaluated to provide various actors with knowledge and support to achieve high-quality services and improved health outcomes for user groups? (Themes 1 and 2)
- How user and stakeholder needs and involvement can be applied to overcome barriers in the coproduction of health innovations—in the spectrum from problem elicitation to implementation? (Themes 1 and 2)
- How knowledge and understanding from private and public stakeholders can improve the capacity for quality improvement and innovation in health and welfare? (Themes 2 and 3)
- How can the multidimensional effects and values of innovations in health and welfare be evaluated on individual and system levels? (Themes 2 and 3)
- How health innovations can be integrated into business models and long-term strategies within private and public sectors? (Themes 1 and 3)
- How private and public partners can collaborate to implement digital technologies for sustainable adoption and diffusion within health and welfare? (Themes 1-3)

The most important goal of the research school is the opportunity for participating partners to create expert competence in areas directly relevant for their business. The more long-term goal for the partners is to increase competence and capacity for research on the development, implementation, and dissemination of digital health technology solutions.

### Established Relationships With Partners From Industry and the Public Sector for Joint Research Training Projects

In the research school formation, it was decided that all doctoral students should be employed by the partner industry or public sector organization, and that at least one senior mentor should be tied to the project. The partners could then choose whether to have additional members active in the research school network.

An essential part of the uniqueness of the research school is related to both industry and public sector being part of the constellation. It is otherwise difficult for the public and private sectors to get access to each other’s needs and demands, which is crucial for both parts to be successful in the digital transformation of health care. By having these different types of organizations represented in the research school network, activities can focus on raising important questions related to the challenges of working within this sector.

Every company will contribute with their specific expertise in their area, which brings a variety of perspectives to the 3 overall themes in the research school and increases the relevance and impact of the research. Some companies are well established within the health care sector, whereas others see this as an opportunity to gain insights into a new market area. The public partners (regions and municipalities) participating in the research school contribute with contextual competence and understanding of the needs, problems, and challenges they face in their operations. In total, the research school comprises partners from 10 companies and 10 public sector organizations, besides the 7 universities.

Further, the research school establishes a network to create personal relationships between the companies’ strategic functions (eg, research and development, managerial levels) and customer representatives (eg, managers of strategic and care departments in the public sector), which are functions that do not often meet in business relation procedures. The research school thereby creates opportunities for participants to discuss common challenges from a strategic or managerial point of view. Integrating these activities within the research school will facilitate learning opportunities for the doctoral students, who are likely to become valuable resources in the companies’ and public organizations’ future strategic work. The partners are responsible for aligning the research questions with challenges relevant to the industry, regions, and municipalities. Their commitments are both to their research project and to that of the research school and they will take part in annual network meetings, providing study visits and contributing to the management of the research school.

### Forms of Collaboration Within the Research School

#### Overview

**Figure 1:**
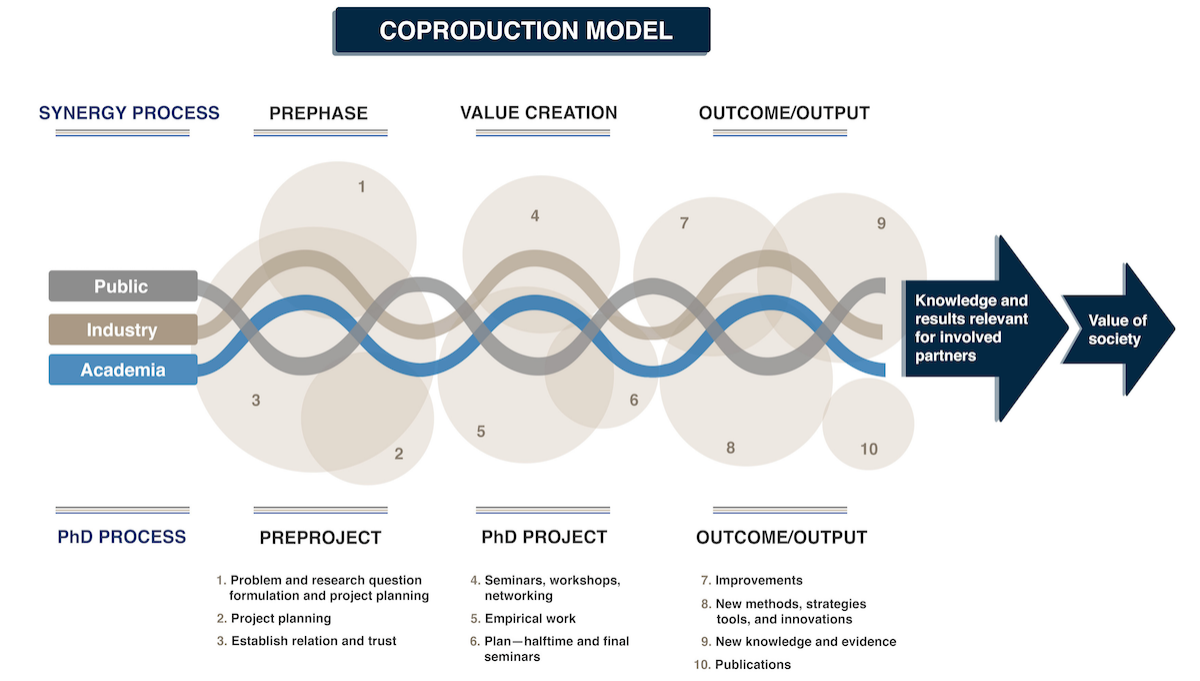
Coproduction model in the research school.

The research school will follow an established process of working in coproduction (Figure 1), which means that researchers and partners come together to share and create knowledge that can be used to face the current digital challenges in health care, while increasing capacity to problem solving in the future. The coproduction approach intends to support learning across partners with the purpose to create new practices for research collaboration that better address real-world problems and mirror the complexities we are facing regarding digitalization in health care.

The model presented in Figure 1 is inspired by Sannö’s model [24] and illustrates how value can be developed throughout the research school process, from the preproject initiatives taken to connect interested parties to a research school, through finding the common themes, needs, and contexts that such a research school wishes to address, and then to actual implementation of the project. The model describes our 2 main processes, both the individual PhD process and the synergy process between partners, within the research school.

Initially, different partners come together to jointly discuss the real-world problem, discuss the key assumptions, prerequisites, purposes and rules of the collaboration, and build trust. In relation to the PhD process, the doctoral students and their supervisors and mentors jointly formulate the problem and research questions as well as design the project. Following this, the value-creation chain for the research school starts, where all partners are engaged in mutual learnings in seminars, workshops, and networking activities. Thus, new knowledge and results for all involved partners are discussed and transferred between partners and integrated to reach new and more comprehensive knowledge in relation to the problem. The doctoral students work on their dissertation project, conduct joint courses within the research school, and participate in seminars and other research activities, in addition to the requirements of their respective doctoral education program. Finally, the comprehensive and solution-oriented knowledge, results, and publications generated in the research school are evaluated in terms of their relevance and impact in relation to the problem being addressed and converted into improvements, new methods, solutions, strategies, and innovations of relevance for included partners.

One of the main tasks of the research school is to arrange joint activities for all partners in the research school, to provide a learning environment focusing on relevant topics related to the scope, the research themes, and the overall research questions of the research school (Table 3).

The aim of the activities is to enrich the progress and training for the doctoral students, as well as strengthening collaboration and coproduction between participating organizations. The legitimacy and relevance of the activities are ensured by creating learning meetings and a learning organization where all participants are actors in the process. Common research questions and results are expected to arise as an outcome of all the different types of activities performed in the research school. The doctoral students’ contribution to a common area of knowledge, in this case related to the 3 themes of the research school, will highlight the expertise that is developed through the research school. By emphasizing the multidisciplinary perspectives that are combined and encouraging contribution to an increased understanding of other perspectives, the knowledge that is created between the different projects goes beyond the scope of the individual project. This is where interesting aspects arise, such as potential synergies and contradictions. To enable impact of this approach, it is of importance to involve both supervisors and mentors, as well as other organization representatives, to take an active part in the research school activities.

**Table 3:** Types of joint activities for all partners in the research school.

Activity	Format	Content	Purpose
Seminars and networking activities	Two-day network activities every 6 months for all participants of the research school (ie, doctoral students, supervisors and mentors, academic stakeholders, company and public organization representatives) ambulating between the participating universities.	Activities will range from lectures to sharing progress reports, seminars, workshops, and study visits. Activities will be designed to meet the partner’s needs and focus on the 3 research themes of the research school.	To give stakeholder groups a chance to meet in various constellations, to identify, develop, and share both specific and generic commonalities and knowledge development.
Doctoral students’ network	Several meetings per year with physical meetings specifically targeted to the doctoral students concurring with international conferences, the joint network days, and the doctoral courses. Digital meetings will be held in between to facilitate continuity in interaction.	Activities will circle around doctoral education domain–specific topics and the 3 themes of the research school and will form a tight constellation among the doctoral students.	To enrich the doctoral students at both a professional and a private level, aiming for connections for future collaborations.
Supervisors and mentor activities	Several meetings per year concurring with the joint network days.	Activities will be in a workshop format and include supervisors and mentors from both university and partners.	To delve into the role as supervisor and mentor, coproduction experiences, scientific course development, and questions on dissemination and impact.

#### International Collaborations

For the research school to be successful it needs to be grounded in international state-of-the-art science and based on cooperative research involving international collaborations. The universities and many of its researchers have established international partners and ongoing research based on existing international networks. The same applies to most of the business and public partners. It is one of the goals of the research school to deliver added value to participating organizations by accessing new, and broadening already existing, networks and striving to further enable and broaden international collaborations. Another goal will be to strive for an understanding of what kind of mechanisms the partners need to be aware of when approaching new markets or diffusing research results and health innovations internationally. The research school will work in several ways to address the internationalization goals. To navigate and receive the best knowledge exchange and outcomes from international collaborations, doctoral students, their supervisors, and the partners need an intercultural and multidisciplinary competency, which will therefore be included as part of the research school’s network activities. The joint network’s meetings and specific seminar sessions will address the topic, and relevant guest speakers will be invited. Another approach will be to encourage and support international mobility, such as study visits to international universities, research institutes, or companies suitable to the contexts of the student’s projects. The students will also be encouraged to participate in international conferences or longer stays at international research institutes, companies, or universities. If possible, a larger group of participants from the research school should aim to jointly attend, thus make a significant impact on the content of a relevant international conference.

#### Organization and Management

As the research school is a collaboration between numerous partners from industry, academia, and public organizations, appropriate project organization and management are crucial to achieve targeted outcomes and set goals for the doctoral students as well as research and educational environments of the participating universities and industry and public partners (Table 4). The management structure aims to strengthen the dialog and transparency between groups and boards when devising, implementing, and assessing strategies and goals in the research school.

**Table 4:** The format, role, and purpose of different groups in the research school.

Group	Format	Role	Purpose
Steering group	Twice per year	Oversees that the research school’s goals and outputs are achieved and suggests actions if required	To provide strategic development advice for the management group and provide support, guidance, and oversight of the progress and direction of the research school
Management group	Every week in the initial phase (first year) and thereafter every second week	Manages all organizational and scientific processes	To coordinate all partners and ensure that the goals of the research school can be achieved at high quality and have an impact
Operational group	Every second week	Executes all activities, such as reports, budget follow-up, routine setup, and communication	To ensure quality control processes and goal fulfillment
Supporting staff	Continuous	Runs local budget administration, local communication, local doctoral student administration, and coordination of tasks related to partner organizations	To assist in execution and securing alignment with the local routines, regulations, and organization of the universities
Scientific advisory board	Once per year. Includes 5-7 senior researchers	Provides advice and support to the management team in scientific issues related to needs, relevance, development, content, focus, and aggregated results and synergies	To represent the themes of the doctoral program
Stakeholder advisory board	Once per year. Includes 5-7 stakeholders	Provides support and guidance by contributing with expertise and experience-based knowledge and skills relevant to the projects	To identify and support opportunities for synergies, dissemination, and implementations

### Format of the Doctoral Education Process

#### Credits for the Doctoral Program

A PhD degree in Sweden requires 4 years of full-time studies and a total of 240 higher education credits. At least half of these credits should be connected to the thesis. The doctoral students within the research school must acquire their PhD degree within 5 years of studies, of which 80% are spent on their studies and the remaining 20% on ordinary organization-specific work tasks.

**Figure 2:**
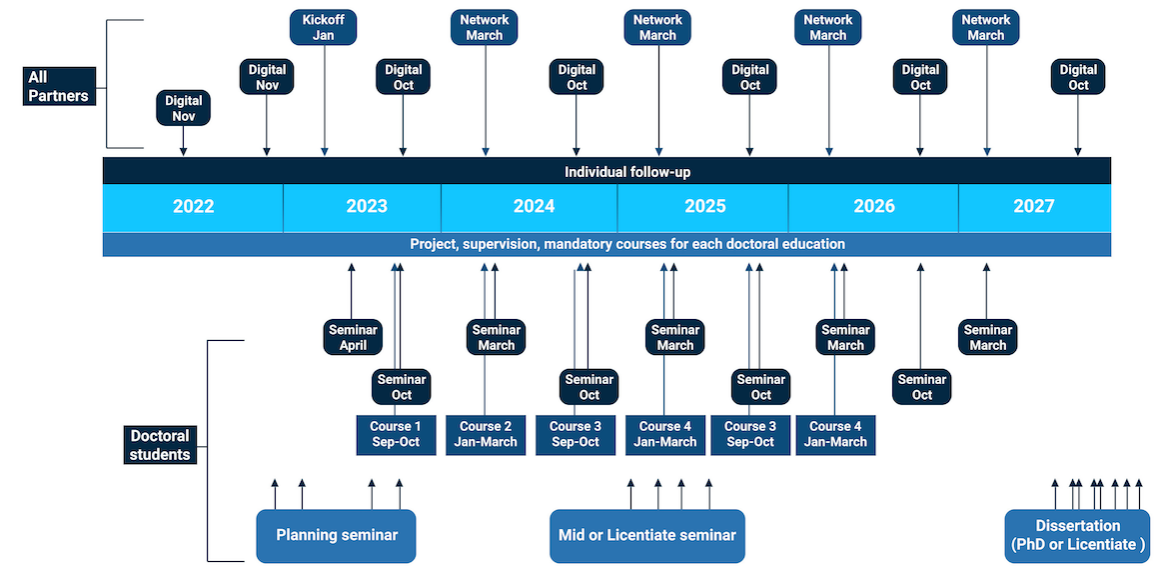
Format of the doctoral education process.

The students within the research school will follow the rules and regulations applied to the respective doctoral education, as specified in the general syllabi. Students participating in the research school, however, can specialize and receive significant added value through the research school activities and the large collaborative network that they are a part of (Figure 2). The research school organizes regular network activities for students, supervisors, and industry and public organization partners, in the form of networking days, consisting of lecturers, seminars, workshops, and study visits. The activities focus to a large extent on creating a collaborative environment between these stakeholders, and facilitate discussions supporting the common goals, challenges, commonalities, and state-of-the-art related to digitalization in the health and health care sectors. This approach should lead to a close contact network between the doctoral students, the cluster of supervisors, partner representatives, and the project organization, which can form support structures related to the doctoral education programs and foundations for further collaboration in other forms. It is, however, important that students participating in the research school are not added an extra amount of workload compared with the fellow doctoral students at their doctoral education programs. Therefore, the supervisory role within the research school is important so that research school activities can be acknowledged as results within the doctoral education, and that compulsory elements in the doctoral education are not duplicated in the research school.

In the doctoral education process, there are several checkpoints (Figure 2). These will be followed up by the research school management group, although formally administered by the university doctoral education. After being admitted to 1 of the doctoral education programs, an individual study plan will be drawn up within the first 3 months and will thereafter be updated once a year. This is the formal document explaining the progress of the student throughout the doctoral education program, and its role and use are highly regulated in the Swedish Higher Education Act (Högskoleförordningen 1993:100). The student’s research proposal plan is also finalized within the first 3 months and revised continuously as the project progresses.

The doctoral students present their project progress on at least three occasions within their doctoral education program (ie, a research proposal seminar, a midterm seminar, and a dissertation seminar), and in at least two other occasions during network activities in the research school (at doctoral and joint network activities; Figure 2). The presentations during network activities have specific focus on the impact of results in the own (or other) organizations or society.

#### Recruitment and Admission of Students

The research school has 2 admissions. The research school allows for a total of 10 industrial doctoral students and 10 doctoral students from regions and municipalities that are publicly funded in the first admission. A potential second admission will add another 10 industrial doctoral students, and again matched by an equal number of students from public sector organizations.

To be admitted to the research school, the doctoral student will be assessed and able to qualify in accordance with the general syllabi at the doctoral education program at the university where the student is to be enrolled. Further, the student will have their employment at the participating company or public sector organization. The doctoral students can be someone already employed at the company but can also be a new recruitment for the specific project. In cases of new recruitments, the recruitment process will be led by the company or public sector organization, with support from the local research school manager and potential supervisors of the admitting university.

The doctoral student will be enrolled in the doctoral education program with the most suitable subject area related to the project. The admitting university is responsible for the administration related to the enrollment of each doctoral student and for making suitable supervisors available for the project.

#### Supervision of Doctoral Students

Each doctoral student has a main supervisor, 1 or 2 assistant supervisors from the universities, and a mentor from the organization where they are employed. If applicable, an assistant supervisor can be affiliated to 1 of the other participating universities to build relations between universities, ensure valuable knowledge transfer, and enrich the project. All supervisors are expected to participate in the research school activities. Mentor candidates from the participating organizations (both business and public) who have a PhD degree and are interested in becoming supervisors will be offered the chance to enroll and participate in the respective university’s supervisory education courses. This education is part of the quality assurance of graduate education and a requirement for being eligible as an academic supervisor.

The team of academic supervisors plays a continuous quality control role in the doctoral student’s educational process, including the study design, empirical and analytical work, research article production, thesis development, and participation in doctoral education courses and research school activities. The supervisory team and the doctoral student agree on the individual study plan, set up milestones, and supervision arrangements including regular and formal progress meetings. The supervision arrangements are formally and regularly followed up within the organization for each doctoral education at each university. It is also reviewed yearly by the project coordinator of the research school, through the individual study plan for the doctoral students and through dialogs with the doctoral students and supervisors.

It is of importance that supervisor roles in the research school are diversely represented with regard to gender and ethnicity. A broad representation will add value to the doctoral students’ perspectives and to the discussion during network activities. It will be the role of the management group to lift these aspects in relation to supervisor appointments.

#### Doctoral Courses

All doctoral educations require a specific amount of compulsory and elective courses, although the number of credits varies between different doctoral educations. The elective courses are chosen to optimize the students’ competence in relation to project needs and interests and should provide a knowledge depth in the field. Students participating in the research school will be offered several small courses (2.5 ETCS credits each) for their elective credits, which are specifically tailored to the research school agenda (Textbox 1).

**Selective courses for elective credits.**

Related to content/research focus:

- Health innovations, application, and value (2.5 credits)
- Coproduction in research and practice (2.5 credits)
- Needs-driven implementation, innovation, and quality improvement (2.5 credits)

Related to skills and abilities:

- Communication of research and value (2.5 credits)
- Academic writing (2.5 credits)
- Ethics in coproduction of health innovations (2.5 credits)

The first course (ie, Health innovations, applications, and value) brings up aspects of innovation with specific relevance to the health and health care context. All courses offered in the research school are developed in collaboration between different universities and disciplines and involve listening to collaborating partners to gather needs. Because of the limited space for further elective courses within the doctoral education programs, further needs for developing shared multidisciplinary knowledge among doctoral students will be accomplished through other research school activities.

### Ethical Considerations

All data and personal data, for both the research school and the separate doctoral projects, will be handled in accordance with the European General Data Protection Regulation (EU) 2016/679. Ethical approval will be obtained from the Ethical Board in Sweden for each doctoral project where this is applicable.

Some ethical considerations could be important to reflect on. For example, ethical challenges can arise in coproduction projects when different perspectives, ambitions, and priorities from stakeholders meet. In the research school, each doctoral project involves 1 doctoral student from either company/region or municipality, 2 supervisors from 1 or 2 universities with expertise in different areas, and 1 supervisor or mentor from either company/region or municipality. The coproduction approach highlights the importance of respecting the perspectives and values of all stakeholders involved in a project [23]. However, while acknowledging that a researcher’s main goal may be to get the results published in scientific journals, it is also necessary to understand that this is not always the goal for other partners. Thus, in each project it is essential to discuss how these issues should be handled to address transparency and common understanding in relation to goals, expectations, and ambitions.

## Results

**Figure 3:**
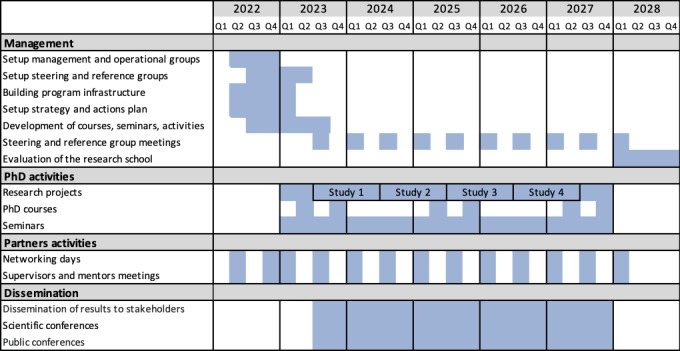
Timeline of the research school.

The research school was funded in December 2021 (Knowledge foundation dnr: 20210047). The research program started in March 2022 and is progressing according to the time plan presented in Figure 3. Briefly, the research school was started between March 2022 and December 2022. During this period, we had set up its infrastructure and the action plans to run the school. Thereafter, the PhD projects will be executed, and these projects will be completed in 5 years. The first group of students have been admitted during autumn 2022 and spring 2023 (in total 15 students). The first results from the PhD projects are expected in 2024 and the first dissertation is expected to be produced in 2026. Additional activities within the research program will include doctoral activities (eg, courses, seminars), networking activities, and dissemination of results.

**Figure 4:**
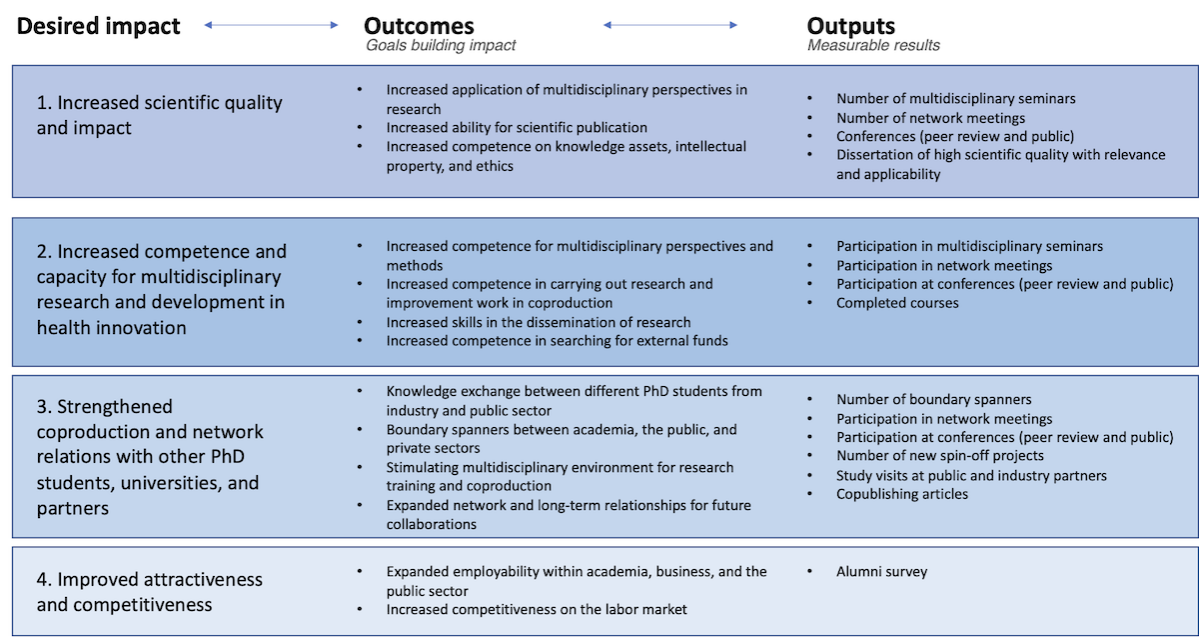
Program logic—PhD students' perspectives.

**Figure 5:**
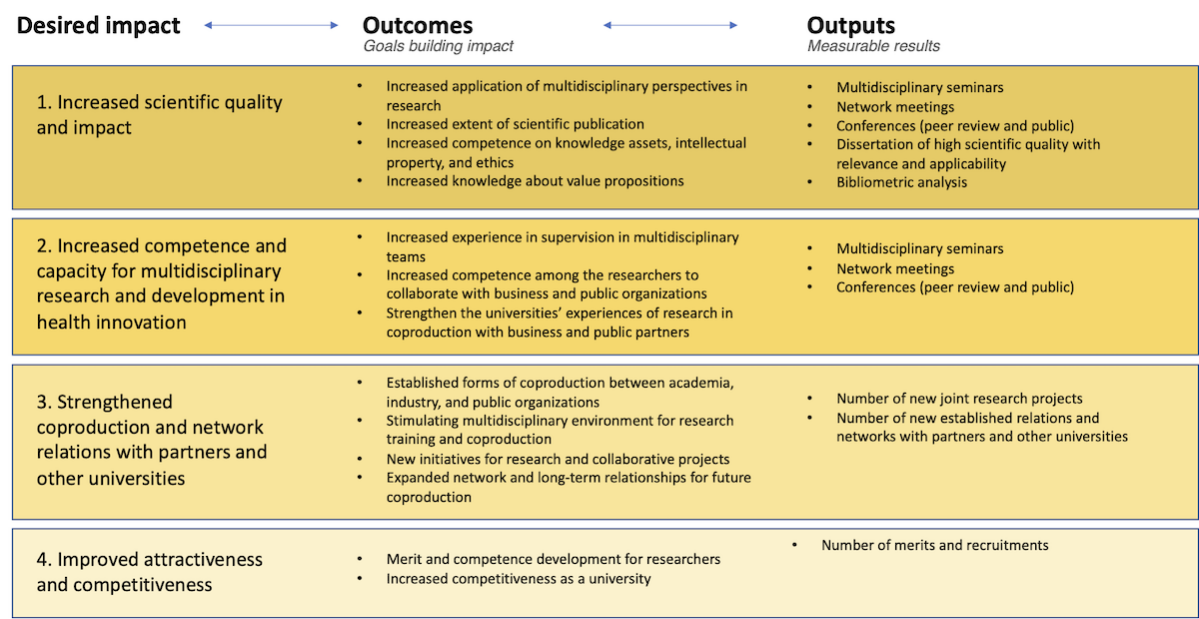
Program logic—universities' perspectives.

**Figure 6:**
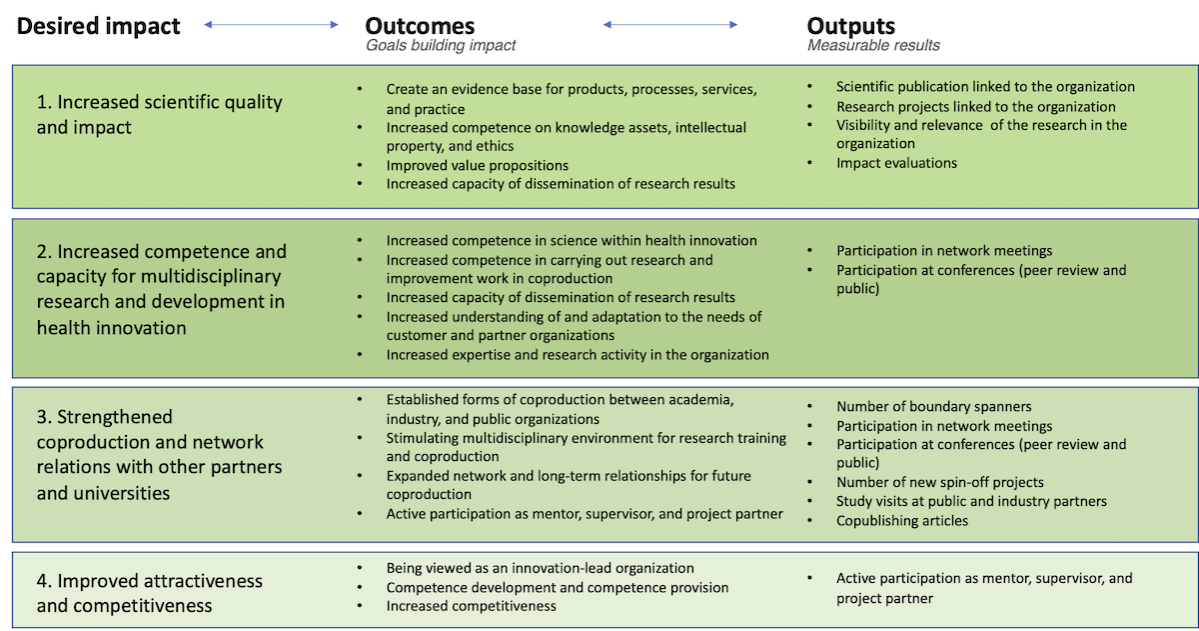
Program logic—company/region/municipalities' perspectives.

To achieve the overall goal, expected outcomes are stated, which contribute to desired impact for the participants involved. To allow for an overview of desired impact and expected outcomes for the different stakeholders in the project, these are presented from the perspective of doctoral students, university and industry, region, and municipalities (Figures 4-6). In the figures outputs, measurable results are also stated.

## Discussion

### Expected Findings

The research school is a collaborative network of higher education institutions and stakeholders from the public and private sector across Sweden. The universities all have a strategic focus on different aspects of health innovation, which together forms a unique constellation of complementary competencies around this topic. By tradition, the health sciences field has extensive experience of collaborating with public sector partners, whereas the engineering and computer sciences field often has more experience in working with industrial partners. By bringing these 2 groups together, the research school is expected to provide a multifaceted added value to the participating partners [25], involving the following:

- Close collaboration with industry or public sector organizations through supervision, project implementation, and research school activities.
- Forming a network with other universities with similar or complementary positioning in the field.
- Facilitation of discussions and plausible collaboration between scholars from different subject areas within the universities (different academic environments).
- Strengthening multidisciplinary research related to health innovation, thus increasing the positioning of the universities, as well as the national academic capacity, in this field.
- Ensuring research of high relevance to industry and public sectors.
- Development of doctoral education programs by integrating industrial doctoral students as well as public sector students.

The benefits of achieving these aspects through an industrial research school are several, and at different systemic levels. For example, the involved academic staff at each university will collaborate closely with the doctoral students and their mentors, which will bring interorganizational and intersectoral understandings and competencies. By developing a better understanding of the health care sector, the shared experiences and perspectives will broaden the horizon and understanding for different research approaches, as well as contextual perspectives [26]. These competencies will be further brought into the research environments and doctoral education programs at each of the universities through the doctoral students, supervisors, and local project coordinators, who act as carriers of this knowledge.

By meeting supervisors from the different participating universities, the research school will facilitate development of supervisory skills, in terms of both insights from adjacent knowledge areas and pedagogic exchange [27]. These aspects of the supervisory role are important to focus on research school activities, to ensure goal fulfillment and relevance related to the supervisors. By being part of a national initiative, rather than a local research school, this effect will have a greater diffusion and impact beyond the academic, business, and public organizations involved in the research school. Further, all involved participants will improve their understanding of the benefits of the synergistic effects resulting from the interaction between academia, industry, and public sector in collaborative development and innovation, as well as the challenges of working interorganizational, which will be problematized within the research school. Another important aspect of the universities’ participation in this research school is related to the capability of keeping up with state-of-the-art within this field. The rapid development of technology and the need for digital technology implementation in the public sector make this industry move very fast [16,28,29]. There is a risk that universities will not be able to keep up with the pace, unless being involved in such development projects. By identifying projects that are of high relevance for industry, research, and public sector, we can ensure that competence and collaborations are being established to continuously keep partners in the forefront of the development. The long-term collaborations formed through the research school also form a great platform for other possible collaborative activities. There are many diverse forms of partnerships between the industrial sector and academia, and the interaction taking place within the research school will open up for new forms of collaborations (eg, student projects in educational programs at the university, a broad pallet of different types of professional research projects, educations for lifelong learning, larger consortiums that strengthen the international positioning within the field, and long-term strategic partnerships).

Despite increased recognition of coproduction in research, there are still demands on research to show its values in practice [23,30]. There is a diversity of coproduction models [31], and how different stakeholders conceptualize, understand, and use research in practice. Most coproduction models that are described and investigated in the scientific literature focus on the collaboration between researchers and users such as health care professionals, citizens, patients, and service users [31], not involving stakeholders from industry. There is evidence that coproduction can lead to benefits and practice change, but there is still a knowledge gap on the success and impact of coproduction between stakeholders from universities, companies, and public actors. Thus, the learnings from this research school are of importance and can tell us something about how coproduction can lead to benefits, value, usability, uptake, and practice change.

### Conclusions

The research school will contribute to a strong development of competence for industry; solve research questions relevant for academia, public sector, and industry; and build stronger ties between the participating organizations promoting value creation, increased competitiveness, and the potential for future collaboration within and outside of the research school partner network. The forms of collaboration between the health care sector, industry, and academia fostered through the research school is vital to build increased competence and to promote knowledge generation and innovation. This requires multidisciplinary constellations that combine the technological development with research and innovation related to stakeholder values and implementation. Succeeding in bringing these perspectives together is a necessity if this advantage in competence is to be applied within the life science sector.

## Appendix

Multimedia Appendix 1 Peer review report from granting agency.

## Abbreviations

CAISR Health: Center for Applied Intelligent Systems Research Health
DHEAR: Digital Health Research
IDC: information-driven care
INFINIT: Research directed towards the generation, quality assurance, analysis and visualization of data using information technology systems and models
LINA: learning in and for the new working life
LOV: learning and caring for sustainable health
RFI: research for innovation
SNAC: Swedish National Study on Ageing and Care

## References

[1] Prince MJ, Wu F, Guo Y, Gutierrez Robledo LM, O'Donnell M, Sullivan R, Yusuf S (2015). The burden of disease in older people and implications for health policy and practice. The Lancet.

[2] Nygren J, Zukauskaite E, Westberg N (2018). User Participation in Coproduction of Health Innovation: Proposal for a Synergy Project. JMIR Res Protoc.

[3] Barlow J, Bayer S, Curry R (2006). Implementing complex innovations in fluid multi-stakeholder environments: Experiences of ‘telecare’. Technovation.

[4] Webster A, Gardner J (2019). Aligning technology and institutional readiness: the adoption of innovation. Technology Analysis & Strategic Management.

[5] Whitelaw S, Pellegrini DM, Mamas MA, Cowie M, Van Spall HGC (2021). Barriers and facilitators of the uptake of digital health technology in cardiovascular care: a systematic scoping review. Eur Heart J Digit Health.

[6] Sachs J (2012). From Millennium Development Goals to Sustainable Development Goals. The Lancet.

[7] Batalden M, Batalden P, Margolis P, Seid M, Armstrong G, Opipari-Arrigan L, Hartung H (2016). Coproduction of healthcare service. BMJ Qual Saf.

[8] Elwyn G, Price A, Wensing M, Grol R, Grimshaw J (2020). Engaging Patients in Healthcare Improvement and Innovation. Improving patient care: The Implementation of Change in Health Care.

[9] Nergårdh A, Andersson L, Eriksson J, Lundberg M, Nordström K, Lindevall M (2018). God och nära vård. En primärvårdsreform. Socialdepartementet.

[10] Jovic V (2018). Svensk sjukvård i internationell jämförelse. Sveriges Kommuner och Landsting.

[11] Nordgren L (2011). Health care matching: conditions for developing a new service system. International Journal of Quality and Service Sciences.

[12] World Health Organization (WHO) (2021). Global strategy on digital health 2020-2025. WHO.

[13] Gray K, Slavotinek J, Dimaguila GL, Choo D (2022). Artificial Intelligence Education for the Health Workforce: Expert Survey of Approaches and Needs. JMIR Med Educ.

[14] Andersson SW, Richardson MX, Cozza M, Lindén M, Redekop K (2021). Addressing evidence in health and welfare technology interventions from different perspectives. Health Policy and Technology.

[15] Ahgren B, Nordgren L (2012). Is choice of care compatible with integrated health care? An exploratory study in Sweden. Int J Health Plann Manage.

[16] (2019). Assessing the impact of digital transformation of health services - Report of the Expert Panel on Effective Ways of Investing in Health (EXPH). European Union.

[17] von Thiele Schwarz U, Nielsen K, Edwards K, Hasson H, Ipsen C, Savage C, Simonsen Abildgaard J, Richter A, Lornudd C, Mazzocato P, Reed JE (2020). How to design, implement and evaluate organizational interventions for maximum impact: the Sigtuna Principles. European Journal of Work and Organizational Psychology.

[18] Vis C, Bührmann Leah, Riper H, Ossebaard HC (2020). Health technology assessment frameworks for eHealth: A systematic review. Int J Technol Assess Health Care.

[19] Greenhalgh T, Wherton J, Papoutsi C, Lynch J, Hughes G, A'Court C, Hinder S, Fahy N, Procter R, Shaw S (2017). Beyond Adoption: A New Framework for Theorizing and Evaluating Nonadoption, Abandonment, and Challenges to the Scale-Up, Spread, and Sustainability of Health and Care Technologies. J Med Internet Res.

[20] Morris Z, Wooding SJ, Grant J (2011). The answer is 17 years, what is the question: understanding time lags in translational research. J R Soc Med.

[21] Nilsen Per (2015). Making sense of implementation theories, models and frameworks. Implement Sci.

[22] Schiavone F, Pluzhnikova A, Invernizzi Ac, Kraus (2021). Digital transformation in healthcare: Analyzing the current state-of-research. Journal of Business Research.

[23] Beckett K, Farr Michelle, Kothari Anita, Wye Lesley, le May Andrée (2018). Embracing complexity and uncertainty to create impact: exploring the processes and transformative potential of co-produced research through development of a social impact model. Health Res Policy Syst.

[24] Sannö A, Öberg Ae, Flores-Garcia E, Jackson M (2019). Increasing the Impact of Industry–Academia Collaboration through Co-Production. TIM Review.

[25] Deming E, Deming E (1994). The New Economics for Industry, Government, Education.

[26] Barlow J (2017). Managing Innovation in Healthcare.

[27] Reed JE, Howe C, Doyle C, Bell D (2019). Successful Healthcare Improvements From Translating Evidence in complex systems (SHIFT-Evidence): simple rules to guide practice and research. Int J Qual Health Care.

[28] Baylan I, Ernkrans M, Hallengren L (2020). Sweden's national life science strategy. Government Office of Sweden.

[29] (2017). För ett hållbart digitaliserat Sverige - en digitaliseringsstrategi. Regeringskansliet.

[30] Smith H, Budworth L, Grindey C, Hague I, Hamer N, Kislov R, van der Graaf P, Langley J (2022). Co-production practice and future research priorities in United Kingdom-funded applied health research: a scoping review. Health Res Policy Syst.

[31] Masterson D, Areskoug Josefsson K, Robert G, Nylander E, Kjellström Sofia (2022). Mapping definitions of co-production and co-design in health and social care: A systematic scoping review providing lessons for the future. Health Expect.

