# Supplementary material for: Strengthening Digital Transformation and Innovation in the Health Care System: Protocol for the Design and Implementation of a Multidisciplinary National Health Innovation Research School
Source: JMIR Res Protoc. 2023 May 31;12:e46595. doi: 10.2196/46595 (PMC10275513; doi:10.2196/46595)
Supplement: Multimedia Appendix 1 [file resprot_v12i1e46595_app1.pdf]

Diarienr

Datum

2021-12-17 20210047

Högskolan i Halmstad
Rektor Stephen Hwang
Box 823 301 18 Halmstad

Beslut avseende ansökan Nationell forskarskola i Hälsoinnovation

Projektansökan har bedömts av KK-stiftelsens bedömargrupp för Företagsforskarskolor 21 efter de kriterier som angetts i utlysningen, utifrån granskningen av den skriftliga ansökan och hearing. Som ytterligare underlag för bedömningen har utlåtande från internationell expertis inhämtats.

KK-stiftelsens styrelse har vid sitt sammanträde den16 december 2021 beslutat i enlighet med vd:s respektive bedömargruppens rekommendation, att uppdra åt KK-stiftelsens vd att inleda förhandling samt sluta avtal med lärosätet avseende finansiering av er ovanstående ansökan. Notera att projektet beviljats medel först då avtalet är klart och underskrivet av båda parter.

Vi ber er kommunikationsansvarige eller motsvarande att kontakta stiftelsens kommunikationsansvarig Eva Högström, eva.hogstrom@kks.se eller tel 073-712 81 45, för en diskussion om hur projektet gemensamt ska kommuniceras.

Nedan följer bedömargruppens motivering till beslut.

Vetenskaplig kvalitet:
Forskarskolan är inriktad på forskning och utveckling inom det breda området "digital health technology solutions". Mer specifikt syftar skolan till att bidra till kunskapsutveckling inom områden som har att göra med utveckling och implementering av nya lösningar, kvalitetsförbättringar och innovation inom hälsoområdet.

Forskarskolan tar sig an intressanta, svåra och multidisciplinära frågeställningar av stort samhällsintresse. För att uppnå forskarskolans ambitiösa syfte har sju universitet och högskolor, samt ett stort antal samarbetsorganisationer, gått samman i en gemensam ansökan. Dessa forskningsmiljöer ger tillsammans en god vetenskaplig nivå, och det finns flera starka forskningsmiljöer som ingår i satsningen. I ansökan saknas perspektiv från patient och brukare. Vid hearingen framkom att planer finns för att tillgodose den frågeställningen. Det innebär också att man kan behöva fråga sig: Vilka delar i hälso- och sjukvårdsprocessen är inte lämplig att digitalisera? Även om planen är ambitiös så förefaller den vara realistisk, en uppfattning som förstärktes vid hearing.

Nytta för näringslivet:
I ansökan beskrivs nyttan för näringsliv och samhälle på en detaljerad nivå. I tillägg till det finns ett imponerande antal avsiktsförklaringar från företag, regioner och kommuner, vars motiv för deltagande är väl beskrivet. Nyttan för varje enskilt företag framstår som god, men det är dock inte lika väl beskrivet hur stor den sammanlagda systemnyttan är. Detta

kompenseras genom en potentiellt mycket stor nytta för den allmänna hälsovården. Om projektets resultat och kunskap kan spridas till ännu fler kommersiella intressenter kan nyttan för samhället bli ännu större.

Förväntade resultat och effekter:
Ansökan innehåller en detaljerad beskrivning av förväntade kort- och långsiktiga mål. Målen är ambitiösa men ibland något oprecist uttryckta. Här hade det varit önskvärt med mer information om hur målen tagits fram samt hur ledningen avser att följa upp dessa. Även om publiceringsstrategin är relativt kortfattad bedöms den som tillräcklig för att kunna dra slutsatsen att forskarskolan kommer att bidra till en positiv utveckling av lärosätenas forskningsmiljöer. När det gäller utbildningsmiljöns utveckling så är beskrivningen fokuserad på forskarutbildningen och där framgår förväntade effekter tydligt. Effekter på grundutbildningsnivån är däremot inte lika väl beskrivna även om det är rimligt att anta att dessa är positiva. Sammantaget bedöms forskarskolan kunna ha en positiv inverkan på lärosätena, initialt framförallt på Högskolan i Halmstad men på sikt också övriga deltagande lärosäten. Samverkan med omgivande samhällsaktörer och mellan de samverkande lärosätena kommer att stärkas betydligt för samtliga medverkande organisationer.

Genomförande:
Genomförandet beskrivs med stor detaljrikedom där det framgår att deltagande företag och övriga samarbetspartners har för avsikt att delta aktivt i forskarskolans verksamhet och bidra till samproduktion. Ledning och organisation är väl beskriven samt är i linje med hur den här typen av verksamhet brukar samordnas. Samtidigt bör det noteras att det stora antalet organisationer från akademi, samt från det omgivande samhället, ställer höga krav på organisations- och samordningsförmåga. Givet forskarskolan komplexitet bedöms tiden som budgeterats för forskarskolans föreståndare något låg. En kritisk framgångsfaktor för en forskarskola är tillvaratagandet av kunskapssynergier. Ett ökat antal gemensamma aktiviteter i nätverket (workshops och särskilt anpassade gemensamma doktorandkurser) bör därför övervägas. När det gäller rollen för den beskrivna "Scientific Advisory Group" är den något knapphändigt beskriven. En utvecklad beskrivning kommer att underlätta rekryteringen av dessa experter.

Sammanfattning:
Forskarskolan avser ett område med mycket stor inom- och utomvetenskaplig relevans. Ansökan beskriver på ett övertygande sätt att uppsatta mål är möjliga att nå samt att dessa kan antas få positiva effekter på deltagande lärosäten, företag och offentlig sektor.

Bedömargruppen rekommenderar projektet att beakta följande kommentarer för att ytterligare stärka forskarskolan:

Utveckla beskrivning av rollen för ”Scientific Advisory Group" för att möjliggöra ett bra urval av experter.

Mer tid bör budgeteras för forskarskolans föreståndare. Det bör även klargöras hur personer som omfattas av cv-katalogen ska bidra till forskarskolans utveckling.

Utveckla planen för gemensamma aktiviteter för att tillvarata kunskapssynergier inom projektet.

Med vänlig hälsning
STIFTELSEN FÖR KUNSKAPS- OCH KOMPETENSUTVECKLING

Error! Filename not specified.

Eva Schelin
Verkställande direktör

Kopia:
Anna Maria Mårtensson
Registrator

STIFTELSEN FÖR KUNSKAPS- OCH KOMPETENSUTVECKLING
Kungsträdgårdsgatan 18, 111 47 Stockholm
Org.nr 802400-4213 www.kks.se
